# Knockdown-Induced Fasting Phenotypes in Flatworms: Insights into Underlying Mechanisms of Feeding Behavior

**DOI:** 10.3390/ijms262411934

**Published:** 2025-12-11

**Authors:** Mikhail Biryukov, Anastasia Dmitrieva, Grigory Chepurnov, Kira S. Zadesenets

**Affiliations:** 1 The Federal Research Center Institute of Cytology and Genetics SB RAS, Lavrentiev Ave., 10, Novosibirsk 630090, Russia; 2 Department of Natural Sciences, Novosibirsk State University, Pirogova Str., 2, Novosibirsk 630090, Russia

**Keywords:** *Macrostomum lignano*, flatworms, stem cells, proliferation, RNA interference, intestine, gene regulation, transcription factor, feeding behavior

## Abstract

The intestine is a multifunctional organ responsible for digestion, nutrient absorption, metabolic regulation, and innate immunity. In flatworms, recent studies have highlighted the importance of intestine-enriched genes expressed strongly in cells of the digestive tract. These genes are not only involved in digestion, nutrient uptake, transport, metabolism, and feeding behavior, but also in the modulating dynamics of stem cells (neoblasts). In *Macrostomum lignano*, the molecular mechanisms regulating interaction between digestive and neural processes remain poorly understood, as in other free-living flatworms. Therefore, identifying the genes required for intestinal integrity and feeding behavior is essential for understanding the underpinning mechanisms. In this study, we examined intestine-enriched candidate genes predicted to be involved in cell differentiation and maintenance of the intestine in *M. lignano* and whether the knockdown of these genes affects other tissues’ functioning. Using RNAi-mediated gene silencing, we identified four genes (*kri1*, *wbp2nl*, *Mlig-tuf1*, and *Mlig-tuf2*) whose knockdown causes pronounced phenotypes, including reduced feeding, fasting behavior, decreased body size and cell proliferation, low reproduction, and altered expression of an intestine-specific apob promoter. We have characterized their roles in intestinal homeostasis and neoblast dynamics and discussed potential mechanisms linking gene disruption to changes in feeding behavior.

## 1. Introduction

The intestine is a multifunctional organ responsible for digestion, nutrient absorption, metabolic regulation, and innate immunity. The digestive system of flatworms is typically incomplete, consisting of a single opening (a mouth), a muscular pharynx, and a branched intestine that distributes nutrients throughout the body [1].

In flatworms, gene-specific silencing by RNA interference (RNAi) has become a powerful tool for studying the function of unknown genes. By selectively silencing tissue-specific genes, researchers can assess how specific molecular components contribute to intertissue regulation of key physiological processes. For example, knockdown of transcription factors controlling goblet cell differentiation in planarians results in a reduced willingness to feed and decreased viability, despite minimal effects on other intestinal or non-intestinal cell types [2]. Similarly, silencing genes involved in nutrient sensing or lipid processing can lead to decreased feeding frequency, impaired peristalsis, or prolonged fasting states, revealing critical pathways that maintain hunger and satiety responses.

Recent studies in flatworm models have highlighted the importance of intestine-enriched genes expressed strongly in the cells of the digestive tract. These genes are not only involved in digestion, nutrient uptake, lipid transport, metabolic signaling, and feeding behavior, but also in the proliferation of stem cells.

The intestine may also play a niche-like role in modulating neoblast dynamics [2]. Neoblasts, the pluripotent stem cells in flatworms, facilitate tissue homeostasis and regeneration of lost or damaged tissues through the initial formation of a blastema (a mass of non-differentiated cells in a wound region). In *Schmidtea mediterranea*, knockdown of several intestine-enriched transcription factors (*nkx2.2*, *gata4/5/6-1*) causes reduced blastema formation and/or decreased neoblast proliferation [2,3]. The intestine-enriched apolipoprotein b is required for not only the transport of lipids from the intestine to neoblasts and their progenitors but also their differentiation and regeneration [4]. Similarly, silencing intestine-enriched HECT E3 ubiquitin ligase wwp1 causes disruption of intestinal integrity, reduced blastema formation, and even neoblast loss [5]. Disruption of intestinal regulatory pathways can therefore lead to altered feeding behavior, fasting-like phenotypes, and overall influence on organismal homeostasis. The planarian intestine is a prominent organ with branched morphology, simple cellular composition [2], and a likely cell non-autonomous role in neoblast regulation, making it a compelling model for addressing fundamental mechanisms of regeneration [6,7]. The pre-existing tissue or blastema undergoes extensive remodeling and rescaling through both migration of cells in undamaged tissues and balanced loss of cells through apoptosis [8] that provide coordinated processes of re-establishment of proportion and function of damaged organs or tissues. The latter, among other things, are regulated by intestine-enriched genes.

*Macrostomum lignano* is known as a model object for regeneration and aging studies for its high ability to regenerate all its cell types with some exceptions [9,10,11,12,13]. This capacity makes it possible for researchers to perform experiments with re-growing tissues of the same worm multiple times. In comparison with other regenerative flatworms such as planarian species such as *Dugesia* spp. [14,15,16] or *Schmidtea* spp., its life cycle from a fertilized one-cell egg to adult worm takes about 2–3 weeks (against 2–3 months for planarians), and its regeneration capacity is limited enough to not produce such mistakes as multi-headed, wrongly regenerated planarians [12,13,17]. *S. mediterranea* exhibits two distinct reproduction modes: sexual through cross-fertilization and asexual reproduction by transverse fission [18]. The latter may limit the production of eggs under experimental conditions. Together with permanent egg production independent from stages and age, *M. lignano* is a more prospective object.

In *M. lignano*, the branched intestinal architecture enables bidirectional flow and coordinated peristalsis. Despite the advantages of *M. lignano* as a model organism, the neural and molecular mechanisms regulating these processes remain poorly understood as in other free-living flatworms. Identifying the genes required for intestinal integrity and feeding behavior is essential for understanding how digestive function is regulated in this organism.

In this study, we examine intestine-enriched candidate genes predicted to be involved in cell differentiation and maintenance of the intestine in *M. lignano* and whether the knockdown of these genes affects other tissues’ functioning. Using RNAi-mediated gene silencing, we identify four genes (*kri1*, *wbp2nl*, *Mlig-tuf1*, and *Mlig-tuf2*) whose knockdown causes pronounced phenotypes, including reduced feeding, fasting behavior, decreased body size, low reproduction, and altered expression of an intestine-specific apob promoter. We characterize their roles in intestinal homeostasis and discuss potential mechanisms linking gene disruption to changes in feeding behavior.

## 2. Results

RNAi-mediated knockdown of most of the candidate transcripts shows no phenotypic effects. Only silencing 4 out of 15 candidate genes showed differences relative to the control RNAi group of worms. Three to four transcripts were found for each gene, namely: (1) *Mlig-kri1-like* gene, homolog of human *kri1* (*Mlig022427.g1*, *Mlig022427.g9*, and *Mlig033815.g9*); (2) species-specific *M. lignano* gene with unknown function (*Mlig013567.g1*, *Mlig025463.g4*, and *Mlig028914.g1*); (3) species-specific *M. lignano* gene with unknown function (*Mlig020807.g2*, *Mlig020807.g3*, *Mlig020807.g4*, and *Mlig020807.g5*); (4) *Mlig-wbp2nl-like* gene, homolog of human *wbp2nl* (*Mlig001691.g1*, *Mlig002860.g3*, and *Mlig022465.g2*) (Table S1). The presented transcripts for these genes have about 96–98% of identity with nearby 100% coverage of their exons (alignments for each gene are presented in Supplementary Materials as Files S1–S4). Here and further for simplicity, we will call them “genes”: *Mlig-kri1-like* as *Mlig-kri1*, *Mlig-tuf1* and *Mlig-tuf2* (species-specific *M. lignano* transcripts of unknown function, 1 and 2, respectively), and *Mlig-wbp2nl-like* as *Mlig-wbp2nl*. We observed phenotypes elicited by the RNAi knockdown of these genes (Tables S1 and S2) in two months after RNAi.

### 2.1. Loss of Function of Target Genes Disrupts Homeostasis in the M. lignano Worms

First, we examined the morphological and behavioral changes: (1) round-circle movement indicating stress-affected conditions for worms, and the main phenotypic indicators of starvation, which include (2) loss of gonads, (3) reduction in worm size, and (4) decreasing offspring production.

Compared to the control RNAi group, we found two prominent differences: an empty digestive tract in the presence of algae in the culture medium and a reduced level of offspring production after a 30-day RNAi on four target genes.

While there were no less than 10 hatchlings in control wells almost every week, experimental wells presented no more than 5 hatchlings (median-2) per well at the fourth week, meaning reliability of the difference between the control and experimental groups (Figure S1, Table S3). It correlates to the time of the knockdown activation processes at the end of the second week as it is supposed to be according to well-known results of previous studies [19,20].

The reduction in the size of worms after RNAi appears to be a consequence of starvation. Considering the possible food insufficiency and existence of different karyotype variants (i.e., varying copy numbers of target genes due to the varying copy number of the large chromosome) in *M. lignano*, we conducted the morphometric analysis on the DV1_8 and DV1_10 worms to account for differences in morphometric traits among worms with varying copy numbers of large chromosomes (two vs. four). To show the changes from possible starvation we assessed the main measurements of the DV1_8 and DV1_10 worms before and after four-week RNAi experiments.

We found that, in comparison to the control RNAi groups, all experimental groups of worms differed by means of morphometric traits (Table 1 and Table S4). For length, the only non-reliable pair was the DV1_8 control and *Mlig-kri1*(RNAi) worms. For width, no changes were found between the DV1_10 control and its experimental groups. In the area of gonads in worms, only the DV1_8 *Mlig-kri1*(RNAi) worms showed no difference relative to the control, and no significant difference in body-worm area was detected between the DV1_10 *Mlig-wbp2nl*(RNAi) and its control. Altogether, in all experimental groups, we observed a decrease of at least two of four measurements due to the RNAi-associated starvation.

### 2.2. Silencing of the Target Genes Mainly Result in Reduced Cell Proliferation

**Figure 1:**
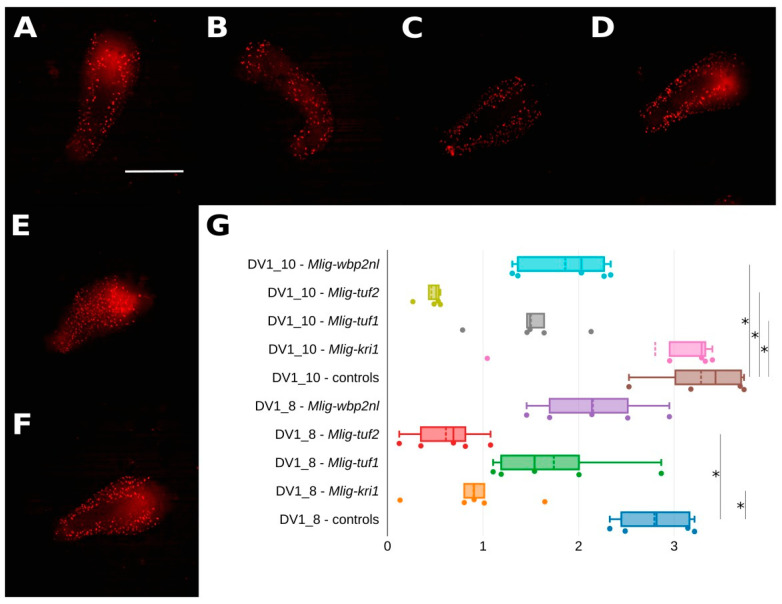
Silencing of the target genes mainly results in reduced cell proliferation. (**A**–**F**) EdU-staining showing cell proliferation in *Mlig-kri1*(RNAi) (**A**), *Mlig-tuf1*(RNAi) (**B**), *Mlig-tuf2*(RNAi) (**C**), *Mlig-wbp2nl*(RNAi) (**D**), and control (**E**,**F**) groups of worms (DV1_8 and DV1_10 controls). EdU labeled cells are in red. Scale bar 300 µm. (**G**) Quantification of EdU-specific fluorescence in RNAi-treated worms. * marked significant difference (*p* < 0.05).

To test the hypothesis of interruptions in dividing cells in the bodies of worms we stained them with 5-Ethynyl-2′-deoxyuridine (EdU) and additionally measured fluorescence intensity. It showed partial decrease in all examined specimens, but its decreasing was non-significant for three pairs, namely, in the DV1_8 line: control vs. *Mlig-tuf1*(RNAi), control vs. *Mlig-wbp2nl*(RNAi), and the DV1_10 line: control vs. *Mlig-kri1*(RNAi) (Figure 1, Table S5). Totally, not all experimental groups represent significance in both DV1 sublines, but each knockdown group showed a decrease at least in one of two sublines. Autofluorescence in the pharynx without clearly defined cells was excluded from the analysis.

**Figure 2:**
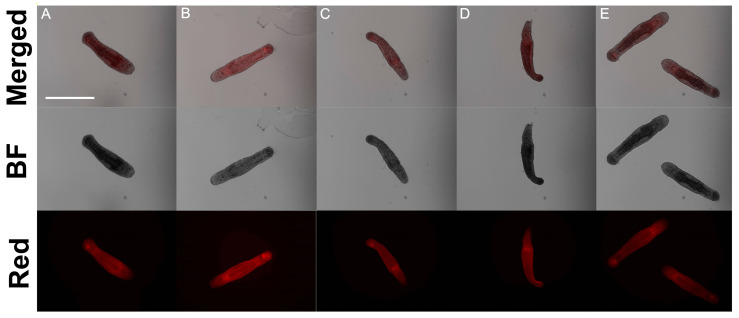
Phalloidin-staining of control RNAi (**A**), *Mlig-kri1*(RNAi) (**B**), *Mlig-tuf1*(RNAi) (**C**), *Mlig-tuf2*(RNAi) (**D**), *Mlig-wbp2nl*(RNAi) (**E**) groups of worms. F-actin filaments are in red. Scale bar 300 µm.

However, phalloidin-staining showed no difference in both patterns and fluorescence intensity in the control and experimental groups of worms (Figure 2 and Figure S2). In each examined specimen, all the main structures are presented: the muscle bag all over the worm with two regions with additional muscle density—the pharynx, which requires more muscles for swallowing, and part of the posterior (tail) region containing seminal vesicles, stylet, and attendant muscles.

### 2.3. RNAi Triggers Silencing of the Transgene Encoding GFP

**Figure 3:**
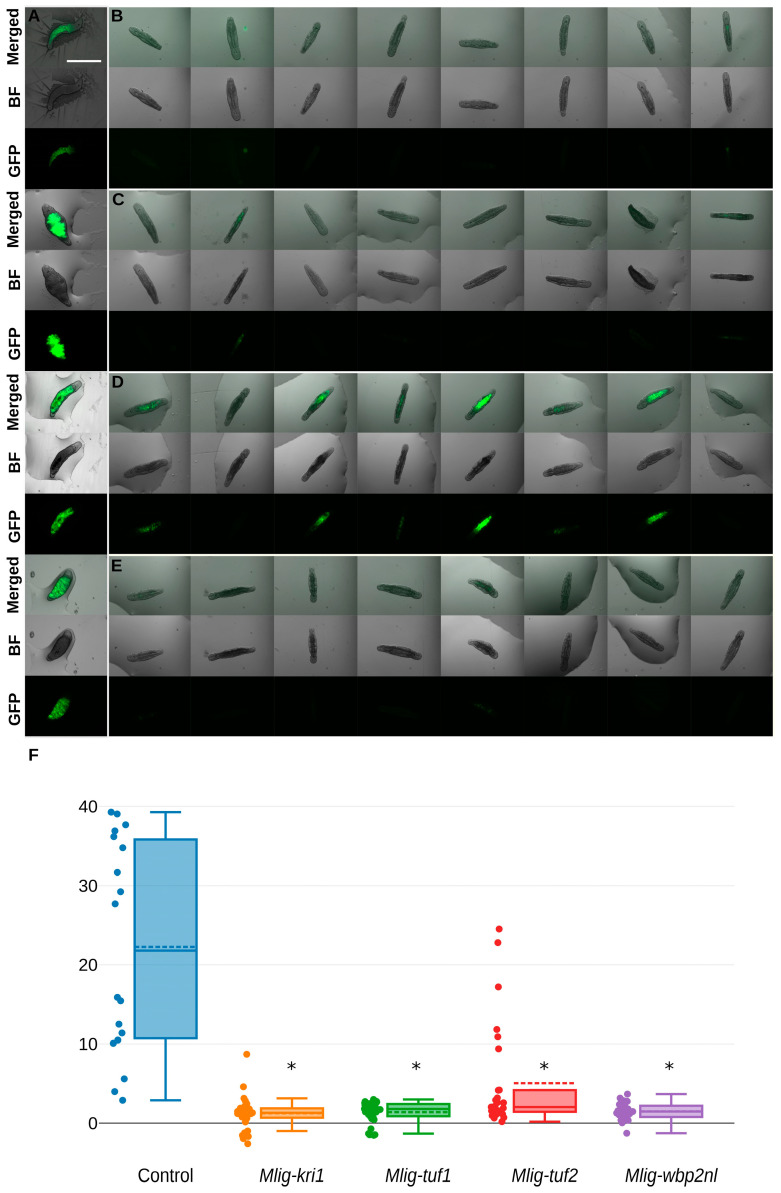
RNAi-mediated loss-of-fluorescence patterns in the *M. lignano* specimens from the transgenic line APOB::GFP (**A**–**E**). Scale bar 1 mm. (**A**) control; (**B**) *Mlig-kri1*(RNAi); (**C**) *Mlig-tuf1*(RNAi); (**D**) *Mlig-tuf2*(RNAi); (**E**) *Mlig-wbp2nl*(RNAi); (**F**) quantification of GFP-specific fluorescence in the RNAi-treated worms. Fluorescence of GFP signals in all target genes decreased to almost zero level. Values below zero resulted from unspecific fluorescence in empty regions taken as background signals. * marked significant difference (*p* < 0.05) compared to control group.

Expecting changes in the digestive tract, we performed knockdown experiment replicates on the transgenic APOB::GFP worms that normally show a specific green GFP fluorescence for the digestive tract of *M. lignano*. According to the published data, the *apob* gene exhibits gut-associated expression [21,22,23], i.e., specific fluorescent signals are found in differentiated and differentiating cells of the intestine. A decrease in the fluorescence level will mean some kind of dysregulation of transgene expression in intestinal cells. In all RNAi-treated worms, GFP expression was reduced relative to the control RNAi group. In all RNAi experimental groups, we observed a significant decrease in GFP fluorescence until the final loss. However, RNAi on the only gene (*Mlig-tuf2*) has shown only partial loss in two of three groups of worms (Table S6). Partial or full loss of the transgene activity regulated by the *apob* gene promoter is presented in Figure 3.

### 2.4. Gene Knockdown Validation

An efficiency of gene knockdown by soaking animals in three out of four target genes by dsRNA-containing medium was confirmed by RT-qPCR. For one gene, *Mlig-tuf2*, we observed the expression level decrease but without reliable values (Figure S3). It correlates with partial loss-of-signal phenotype in the transgenic line, APOB::GFP, meaning incomplete downregulation of the knocked down gene

## 3. Discussion

The tissue architecture of flatworms and the mode of cell turnover during development, homeostasis, and regeneration is unique within the animal kingdom. In *M. lignano*, all cells, including the germ line, are derived from somatic cells called neoblasts. The fact that the molecular regulation of stem cells and the germ line appears to be conserved makes flatworms suitable organisms to study questions of stem cell biology. Previously, different studies on target genes involved in the maintenance of neoblasts and some cell-specific types (e.g., neoblasts, adhesive and reproductive organs, etc.) were conducted, and the essential genes specific to *M. lignano* were defined [19,24,25,26,27,28,29,30,31,32]. It should be noted that in certain instances, after conducting studies on *M. lignano*, the predicted role of function of the candidate gene was not approved and was unexpectedly linked to another function [25,28]. In this study, we examined a few candidate genes predicted as probably important for maintenance of the digestive tract of *M. lignano*.

### 3.1. Visual Changes Indicates Traits of Starvation

First observations in the knockdown worm groups, *Mlig-kri1*(RNAi), *Mlig-tuf1*(RNAi), *Mlig-tuf2*(RNAi), and *Mlig-wbp2nl*(RNAi), have shown a decrease in a number of common vital traits. First, at least two of four morphometric characteristics being measured (worm length and width, area of whole worm and its gonads after being flattened) had values less than the control RNAi groups. In most studies of flatworms, such a tendency is suggested to be either rejuvenation as an outcome of starvation or starvation itself [33,34]. Secondly, the reduced offspring production can be explained by disruption of the normal functioning of gonads and/or insufficiency of nutrient resources. The absence of algae in the digestive tracts of worms in the experimental groups makes starvation a more plausible consequence of RNAi-mediated silencing of the targeted genes. The described phenotypic changes are consequences of long-term starvation in flatworms [35] that lead to the decrease in size of worms, regression of their reproductive system, and reduced mitotic activity of the neoblasts [17].

### 3.2. Decreasing Cell Divisions Reveal Complex Disrupting Effects of Gene Knockdown

To confirm the hypothesis of RNAi-induced starvation we examined the possible changes in amounts of cell divisions. Visually, all groups of worms were characterized by the similar distribution of dividing cells. In the RNAi groups the measured fluorescence from positively stained cells decreased in comparison to the control groups. Together with all abovementioned reduced characteristics, a decrease in activity of cell divisions may affirm that worms are suffering from starvation [35].

However, the underlying processes of starvation after knockdowns of target genes remains unclear. We considered two possibilities. First, starvation could be the direct result of knockdown in each gene disrupting undisclosed feeding-associated processes. Second, RNAi leads to severe large-scale effects on the homeostasis of worms, including the activity of dividing cells. A decrease in dividing cells may result in ineffective cell renewal in the intestine followed by inability to feed.

### 3.3. Loss-of-Signal in the Gut-Specific Transgenic Line Exhibits Termination of Intestine Activity

We perform additional RNAi experiments on the transgenic line APOB::GFP that marks intestinal cells in *M. lignano* to detect possible changes in the intestine. Normally, all differentiated and at least part of differentiating intestinal cells would be fluorescent. We found that almost all cells with active *apob* promoter were affected as their fluorescence has gone. With the exclusion of the non-target RNAi effect on GFP activity shown by plasmid constructions verified by Sanger sequencing, the main explanation is disruption in the key processes of *apob*-related pathways. As long as *apob* is known as being strongly associated with the intestine, the use of the transgenic line APOB::GFP confirmed that the silencing of all target genes finally affects maintenance of intestine homeostasis.

### 3.4. Muscle-Specific Staining Shows No Signs of Mechanical Inability to Feed

We suspected that some changes could be started not from disruptions in complex pathway regulation processes but because no food comes through the pharynx, leading to all starvation signs. Commonly, the phalloidin-staining procedure shows if there are some unnatural patterns of muscle tissue organization [36]. No observed differences in the muscle grid make a scenario of inability to intake food due to muscle dysfunction less probable. However, the observed phenotypic effects could be caused by other factors. As an example, it may result from avoiding algae as a part of disgust-of-eating behavior.

### 3.5. Scenario Consideration of the Target Genes Knockdown Effects

According to all our results, we still cannot certainly say (a) if knockdowns lead to primary disruption resulting in starvation followed by all other described phenotypic observations, or (b) if the knockdown procedure for each target gene interrupt plenty of downstream processes leading to all observed changes, including induced starvation? That is why we will discuss the main interpretations of our findings.

#### 3.5.1. Muscles Seems Not Involved Directly in Worm Starvation

The idea of muscle dysfunction making the gut incapable of containing or even swallow food should be rejected. With an indistinguishable difference between experimental and control groups muscle patterns we are unable to lean on an interpretation of feeding inability based on mechanical reasons.

#### 3.5.2. Neural System Still Could Play with Hunger

In addition to mechanical reasons, the behavioristic worm inability to feed should be debated. The feeding behavior process is complex and can be categorized into four sequential phases: initiation of feeding motivation, search for food sources, recognition of nutritional quality, and food intake (capture and swallowing). The digestive tract of most flatworms is incomplete, with an opening, “the mouth”, that is also used to expel digestive system wastes, pharynx, and intestine. The special organization of the *M. lignano* branched digestive system provides bidirectional flow and sequential peristalsis. That is why we need to hypothesize at least the existence of some neurointestinal signaling that regulates worm activity and behavior in food preying and digestion. The role of the nervous system in regulating feeding, including bidirectional flow in branched intestines of flatworms, is not clear. Therefore, with all our findings, we cannot decline or insist on such a hypothesis, but it should be reviewed further.

#### 3.5.3. Target Genes Are Essential for Intestine Functioning in *M. lignano*

Whatever happens first after RNAi, intestinal cells are one of the main targets. Our result of loss of GFP signal allows us to prove that the intestine either fully or partially (at least for *Mlig-tuf2*) stopped digestion processes. All other observed phenotypic manifestations can be considered as consequences of digestion-stopped starvation. The main question is, where did the changes start? It suggests others as well: which cell population is affected first? What process is involved directly? What happens with affected cells?

We can argue that our target genes play a role as TFs or related genes. A likely realistic hypothesis is that genes predicted as intestine-enriched actually play an unknown role in its homeostasis. Loss of function in the *apob* promoter region, which plays the role of a TF planting site, means no existence of active proteins that normally act with the current region. This way, the knockdowns of candidate genes should happen somewhere upstream of protein activity of *apob* in the regulative gene network. Under this scenario, our gene candidates are key participants of cell regulation pathways.

On the other hand, the decreased amount of the dividing cells throughout the worm can be discussed as a primary factor. It seems quite understandable that a general decrease in dividing cells leads to less renewal of the organ, followed by its aging, and, ultimately, to the cessation of its functioning. As a result, the observed signs of starvation occur, i.e., a decrease in the number of proliferating cells, worm size features, and fertility [35].

Next, we also should notice how well the hypothesis of interruptions in neurointestinal signaling fits our results. It is believed that homeostasis maintenance in flatworms is based on unclear intercellular intermediates transmitting signals between neoblasts and their microenvironment, suppressing or activating their differentiation. Additionally, there should be hungry-or-fed signals regulating food searching and intake activities. We consider the possibility that the RNAi of target genes could also disrupt molecular signaling, without which both of these processes could be disrupted with the following signs of starvation.

### 3.6. Target Genes: GO Terms and Data from RNAi Assays on Other Worm Models

Summarizing all the mentioned options, we discussed the main possibilities of processes that could be interrupted by target gene knockdown: (1) high-ranking regulation processes of signaling pathways, including even transcription factors or genes they interact with; (2) genes involved in regulation of neoblasts and their dividing activity; (3) signaling molecules either between the intestine and neural system or (4) between the intestine and neoblasts.

To shed light on the functions of the target genes and explore their potential pathways, we analyzed them in terms of Gene Ontology (GO) for previously described and predicted information to known homologs in worm species, such as *Caenorhabditis elegans* and *S. mediterranea*.

#### 3.6.1. *Kri1*

According to the database, *kri1* is predicted as a zinc finger gene (“zinc finger, zz type pthr14490” annotation links to PANTHER [37]), which localizes its activity mostly in the nucleus and is involved in RNA biosynthesis and metabolism and early stages of ribosome assembly. Restriction in the ITS1 region is necessary for 5.8S RNA separation from future 18S RNA needed for further ribosome existence [38]. *kri1* was identified as a plausible pre-rRNA processing factor [39,40] and then found taking place in apoptosis and cell arrest processes [41], autophagy [42], and even intestinal bleeding in cases of severe anemia [43]. Knockdown of *Mlig-kri1* may lead to loss of the number of dividing cells directly by being involved in autophagy processes. This gene seems to be a multifunctional one involved in all previously described molecular mechanisms.

In *C. elegans*, *kri1* is expressed in the intestine and pharynx throughout all stages of postembryonic development and adulthood [44]. They have shown that germline removal leads to the extended lifespan in *C. elegans* via triggering the nuclear localization and activation of the DAF-16/FoxO transcription factor in the intestine [45]. Taking into account the signaling from the reproductive system to the intestine since the transcriptional factor DAF-16 accumulates in intestinal nuclei and functions in the intestine, Berman and Kenyon suggested that the germline communicates with the intestine via lipophilic-hormone signaling and that the response to this signaling in the intestine requires the protein KRI1. In principle, *kri1* could act in the reproductive system, the intestine, or another tissue to influence lifespan. They found that in their self-created transgenic KRI1::GFP *C. elegans*, the *kri1* promoter was expressed specifically in the intestine and pharynx. This fusion was able to extend the lifespan of a *kri1* mutant. Given the demonstrated importance of the intestine in this pathway, the simplest interpretation of these findings is that *kri1* acts in the intestine to increase lifespan.

#### 3.6.2. *Wbp2nl*

The *wbp2nl* gene is an N-terminal-like copy of *wbp2* that belongs to a group of WW binding domains (“WW domain binding protein 2,” isoform e pthr31606 in PANTHER [37]). Its localization is nucleic and in the perinuclear theca according to direct GO classes, including “transcription coactivator activity”, “nucleus”, “positive regulation of DNA-templated transcription”, “WW domain binding”, “perinuclear theca”, “sperm head and flagellum”, “egg activation”, and “male/female pronucleus assembly”. Therefore, the main predicted functions of *wbp2* are as follows: transcription-associated activity, chromatin and WW domain binding processes, and interactions of sperm and oocyte [46,47]. We are unable to discuss perinuclear theca sperm-to-oocyte functions according to less fertility and hatchling production in experimental groups; however, the WW site is known as a target for protein-to-protein interactions for a set of signaling and regulative proteins, meaning the gene could take place in multiple processes, including metabolic ones [48].

In the literature, the *wbp2* gene is an emerging oncogene and serves as a node between the signaling protein Wnt and other signaling molecules and pathways [49,50]. It is strongly associated with YAP/TAZ signaling, part of the Hippo pathway acting as a transcriptional coactivator, with its expression and activity being tightly regulated by post-translational modifications, including phosphorylation and ubiquitination, leading to changes in protein–protein interactions, subcellular distribution, and protein degradation [51]. The Hippo signaling pathway mainly participates in the regulation of cell proliferation, fate, and organ growth, while YAP/TAZ controls cellular processes like proliferative activity, apoptosis, cell cycle progression, and organ size by interacting with transcription factors. The Hippo pathway was previously studied in *M. lignano*, as it seems to play a more complex role in highly regenerative organisms, including flatworms. It was shown that after RNAi-derived disruptions in pathway regeneration ability, changes to hyperproliferation and non-accurate regeneration [52] make *wbp2nl* a more interesting target.

We admit some uncovered changes in worms that were not detected by selected methods. Changes in gut tissue after *Mlig-wbp2nl* knockdown should be interpreted as the result of insufficient activity in a number of processes that could be affected by the studied homologous gene. Altogether, this means the gene plays a role in high regulation processes. According to our results, we can state only its regulative nature in *M. lignano*.

#### 3.6.3. *Mlig-tuf1* and *Mlig-tuf2* Lacking Homologs in Other Species

Our two previously undescribed genes were studied by GO terms with an additional search in the Conserved Domain Search (CD-search) tool that finds tertiary protein structure homology with profiles from the Conserved Domain Database (CDD). Results of CD-search requests for both *Mlig-tuf1* and *Mlig-tuf2* are presented in Figure S4.

For one of two other studied genes with no well-described homologs, GO analysis shows only a class of possible proteins as a product of the gene. Both GO terms and CD-search found *Mlig-tuf1* to be a protein with lipase class 3 domain (Lipase_3 in each database; and cd00519 domain, a part of the abhydrolase superfamily, in CDD). The superfamily is attributed to the GO:0016788 term, which is involved in lipase, nuclease, and phosphatase activities. Albeit the description on the superfamily level is not enough to clarify the real process the gene is involved in, according to the main Lipase_3 superfamily gene functions, the gene could take place in regulative roles, matching all the functions discussed about the *kri1* and *wbp2nl* genes.

For *Mlig-tuf2* there was no described GO term, but using the CD-search tool we found two domains with low e-values (meaning some evolutionary distance from current domains and their direct functions). They are known as the domain of cell adhesion molecules (CD05717 in CDD named “IgV_1_Necl_like”—immunoglobulin-like domain of nectin-like molecules) and the domain of CLECT (smart00034 in CDD stands for C-type lectin or carbohydrate-recognition domain), which also runs the molecular binding function. Because both found domains play a role in cell adhesion and binding molecules, this implies the potential involvement of the gene in an unknown kind of interaction associated with a combination of two binding domains diversified from known relatives. It could be a signaling molecule or the one taking a signal via various affinities to one or more molecules.

### 3.7. Neoblast- and Intestine-Enriched TFs in Regenerating Flatworms

The mechanism of how different tissues interact with one another for coordination of aging or regenerative processes is not well understood. Transcription factors play a critical role in cell differentiation in planarians [45]. Moreover, there are some examples of TFs playing a role not only in a tissue they are expressed in.

Forkhead box O (FoxO) proteins are transcription factors that play critical roles in cellular processes such as proliferation, differentiation, and metabolic regulation. Previous research has investigated the role of the *FoxO* gene in *S. mediterranea*, showing that the knockdown of *Smed-FoxO* disrupts tissue homeostasis, resulting in disorganized epidermal cells [45].

*Smed-gata4/5/6*, the homolog of the three mammalian *gata-4, -5, and -6* factors, is expressed at high levels in differentiated gut cells but also at lower levels in neoblast populations. *Smed-gata4/5/6* knockdown results in broad differentiation defects, especially in response to damage, and these defects are not restricted to the intestinal lineage [3].

In planarians, knockdown of an intestine-enriched transcription factor, *nkx2.2*, inhibited neoblast proliferation and formation of the regeneration blastema, the unpigmented mass of tissue produced after amputation. These observations suggested that the intestine could play a non-autonomous role in regulating stem cell dynamics.

In *S. mediterranea*, *apob*(RNAi) has affected the regeneration of organs, including the brain, pharynx, and intestine. Although both the brain and pharynx regenerated, these organs were smaller than in the controls. This suggested that regeneration was delayed, but not blocked, similar to the phenotype for cells expressing polarity cues. Intriguingly, unlike *nkx2.2*(RNAi), *apob* knockdown does not reduce the abundance of phosphoHistone-H3-S10-positive neoblasts, indicating that the proliferative defect in *nkx2.2*(RNAi) animals is not caused by APOB loss, and additional downstream regulators of proliferation remain to be discovered [4].

It could be promising to identify other intestine-enriched transcription factors that could play distinct roles in not only gut cell differentiation and maintenance but also in the nearby cell microenvironment, suggesting multiple possible cell non-autonomous influences on the dynamics of neoblasts. In *S. mediterranea*, knockdown-regulated intestinal TFs *nkx2.2* or *Smed-gata4/5/6* led to reduced proliferation and blastema production [2,3]. It would be interesting to discover intestinal cell-type diversity and identify their enriched transcripts. This could be useful for knowledge on organ regeneration as well as the evolution of patterning mechanisms in bilaterian digestive systems [2].

TFs exhibit pleiotropic roles in tissue regeneration, with their specific functions influenced by different stimuli and cell types.

### 3.8. Further Perspectives

According to insufficient data, genes still need to be studied to clarify their function. We suggest characterizing the intestinal transcriptome using RNA-Seq, bioinformatics tools, whole-mount in situ hybridization, and RNAi. It may reveal underlying and subsequent targets and the processes of candidate genes involved in cellular processes of proliferation, differentiation, metabolism, and stress responses. Additionally, studies of regeneration models such as *M. lignano* are likely to generate new insights into dysregulation of the abovementioned processes underlying human gastrointestinal pathologies associated with aging and disease.

## 4. Materials and Methods

### 4.1. Study Organism

The laboratory lines of the free-living flatworm *Macrostomum lignano* were kept in glass Petri dishes with autoclaved and filtered nutrient-enriched artificial seawater (ASW) at a salinity of 32‰ (Guillard’s f/2 medium according to the protocol [53]), at 20 °C, in a 14:10 hours light/dark day cycle, and fed with the diatom *Nitzschia curvilineata* ad libitum, as described in [17,54].

**Figure 4:**
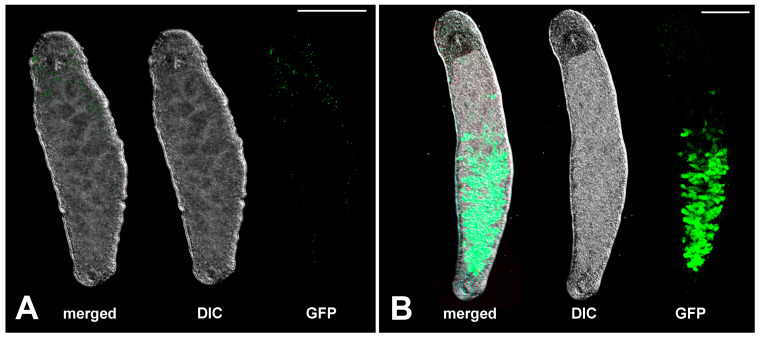
Microimages of alive specimens of *M. lignano* from the wild-type NL12 line (**A**) and the transgenic line APOB::GFP (**B**). DIC—differential interference contrast image of alive worm. GFP-positive signals (green color) in the intestine represent branched gut. Scale bar 200 µm.

In the present study, we used the wild-type line NL12 [17], two pure inbred sublines, DV1_8 and DV1_10 [55], and the transgenic APOB::GFP line [21,56]. The sublines NL12 and the transgenic APOB::GFP line were established in Prof. Berezikov’ group [17,21,56] and were kindly provided by Prof. Eugene Berezikov and currently are cultivated in our laboratory. The DV1_8 and DV1_10 sublines were earlier established in our laboratory [55] and have continued to be cultivated in our laboratory. Based on the karyotype of worm founders (2n = 8 vs. 2n = 10), the DV1_8 and DV1_10 sublines originated from the DV1 inbred line, and their karyotypes differ with the copy number of the large chromosome 1, i.e., two copies in the DV1_8 line (2n = 8) and four copies in the DV1_10 (2n = 10) [55,57]. The transgenic line has a green, fluorescent marker GFP under control of the promoter of the *M. lignano apob* gene homolog (*Mlig005144.g2*). The APOB::GFP::Ef1a_3′UTR transgene [21,56] exhibits gut-specific expression in worms of this line. The structure of *apob*-derived GFP pattern is presented in Figure 4. Before being used in experiments, worms were selected for specific *apob*-directed GFP pattern.

All manipulations with worms were performed under the binocular microscope Altami CM0745 (Altami, Saint-Petersburg, Russia).

### 4.2. Target Gene Selection

Based on previous RNAseq studies [19], there is available genome-guided transcriptome assembly Mlig_3_7_DV1_v3 [29], other https://macgenome.org (last accessed on 3 November 2025) resources, and unpublished RNAseq data. The latter were obtained for GFP-positive cells collected using flow cytometry from dissociated whole worms belonging to the transgenic APOB::GFP line. In a result, the transcripts associated with the intestine were separated from others belonging to other tissues (this work was performed by Prof. Berezikov’ group, and the potential intestine gene transcripts were kindly provided by Eugene Berezikov). From the listed intestine-enriched transcripts, we have chosen 15 candidates with at least five times increased expression as putative targets for further experiments (the list of transcripts is presented in Table S1). Genes were analyzed for Gene Ontology (GO) [58,59] with the following analysis by CD-search [60] for conserved protein domain (from Conserved Domain Database, CDD [61]) in genes which had no relative GO terms.

### 4.3. dsRNA Production and Knockdown Procedure

The production of double-stranded RNA (dsRNA) was performed according to previously published protocols [19,20,28,29] via cloning gene constructions into T444T plasmid with the following production of RNA fraction in the HT115 *Escherichia coli* strain. Gene constructions were obtained by PCR amplification (LR HS-Taq PCR kit, Biolabmix, Novosibirsk, Russia) with the primers (sequences presented in Table S2) from a total RNA of 200 worms reverted by RNAscribe RT kit (Biolabmix, Novosibirsk, Russia). Primers had additional restriction sites, *BglII* and *KpnI*, which were used for cloning in T444T plasmid under T7 promoter regions. Insertions in T444T plasmid were verified by Sanger sequencing on an automated sequencer, ABI PrISM 3100 Avant Genetic Analyzer (Applied Biosystems, Waltham, MA, USA), with a Big Dye terminator sequencing kit (Applied Biosystems, Waltham, MA, USA) at the SB RAS Genomics Core Facility (Novosibirsk, Russia). Production of each gene dsRNA was performed in HT115 *E. coli* which contained modified T444T plasmid cultures in LB by two-step shaking at 370C: first, overnight incubation in small amounts with two antibiotics (for plasmid—ampicillin 100 µg/mL and for HT115 cell line—tetracycline 50 µg/mL) with the following production step in 20 mL volume with ampicillin only.

Candidate genes were knocked down by means of RNA interference (RNAi) with dsRNA delivered by soaking, as previously described [14,19,28,62]. We used approximately 10 animals per well of a 24-well plate for candidate gene RNAi treatment and dsRNA of *heh1* [63], a gene from *C. elegans* with no known *M. lignano* homologs, as a negative control [20]. Twenty-day-old worms were maintained in 24-well plates and incubated with approximately 3 μg of target dsRNA solution (commonly 15–30 μL of 100–200 ng/μL) in f/2 medium containing diatoms (preplanted in wells two days before worms by 200 μL of scraped out algae suspension). The dsRNA solution was changed two times per week.

### 4.4. Genetic Knockdown Validation

To confirm the efficiency of the RNAi knockdown, we performed qRT-PCR using three replicates (10 worms per each replicate) which were treated by either candidate genes or *heh1* dsRNA for four weeks. After that, worms from each replicate were rinsed with a fresh f/2 medium, suspended in 500 μL of TRIzol reagent (Ambion, Austin, TX, USA), and stored at −80 °C. Total RNA was isolated from the samples following the manufacturer’s protocol. The evaluation of RNA integrity was performed using Agilent 2100 Bioanalyzer (Agilent, Santa Clara, CA, USA). The RNA integrity number (RIN) was about seven for all extracted RNA specimens. The RNA was reverse transcribed to generate cDNA using Reverse transcriptase from RNAscribe RT (Biolabmix, Novosibirsk, Russia) with a mix of oligo(dT) and random hexamer primers. The expression of candidate gene mRNAs was checked along with that of three housekeeping genes used in previous studies (*cox5b*, *eif1A*, *gapdh*) [28,62]. The expression level was calculated relative to the geometric mean expression level of three endogenous control genes according to the described method [64]. The primers used are listed in Table S2. Primer efficiency was tested by a series of dilutions of the cDNA template. A melt-curve protocol was performed for each primer pair to detect specific products. qPCR was performed with the BioMaster UDG HS-qPCR Lo-ROX SYBR (2×) (Biolabmix, Novosibirsk, Russia) using the Bio-Rad CFX96 Touch™ (Bio-Rad Laboratories, Hercules, CA, USA). Gene expression data analysis was performed using CFX Manager™ Software version 2.1 (the value of the “threshold” indicator was set to 60).

### 4.5. Phalloidin and EdU Staining

After 3–4 weeks of RNAi treatment, the worms were used for phalloidin-staining of F-actin filaments. The DV1_8 and DV1_10 sublines additionally were stained with EdU (5-Ethynyl-2-deoxyuridine) to detect dividing cells.

Before fixation, worms were relaxed in 2:1 7.14% MgCl_2_/ASW for 30 min at room temperature (RT). Then, worms were fixed in 4% paraformaldehyde (PFA) in 1× PBS for 30 min at RT. Then, the worms were washed in 1× PBS for 5 min at RT, permeabilized in 1× PBST (0.1% Triton X-100 in 1× PBS) for 5 min at RT, and washed in 1× PBS. Phalloidin staining was performed using Phalloidin CruzFluor™ 647 Conjugate (Santa Cruz Biotechnology, Dallas, TX, USA) (1:200; 1% BSA in 1× PBS) for 15–20 min at RT, followed by washing in 1× PBS for 5 min at RT. Then, the stained specimens of *M. lignano* were mounted in VECTASHIELD Antifade Mounting Medium (Vector Laboratories, Newark, CA, USA) [65].

The dividing cells in worms were stained in the EdU (5-Ethynyl-2-deoxyuridine) solution (20 μM in ASW) (Lumiprobe, Moscow, Russia). Detection of EdU-labeled nuclei was performed with Cyanine3 azide in the presence of a copper catalyst according to the manufacturer’s protocol (Lumiprobe, Moscow, Russia).

### 4.6. Microscopy Analysis, Morphometry Measurements, and Fluorescence Intensity Analysis

To assess the possible effect of changing the copy number of the large chromosome 1 (i.e., changing the genome ploidy level) on knockdown of candidate genes, we performed measurements of the main morphometric traits (length, width, body area, and area of gonads) of the DV1_8 and DV1_10 worms before and after RNAi. For that, immobilized squeezed living worms were prepared and examined at the stereomicroscope Axio Zoom.V16 (Zeiss, Jena, Germany) equipped with the digital camera HRm (Zeiss, Jena, Germany). Microimages were captured using the ZEN lite software version 3.0 (Zeiss, Jena, Germany), and morphometric assessment of worms was applied using ImageJ version 1.54p [66,67,68]. Microimages with fluorescent signals were captured at the same settings for the further measurements of fluorescence intensity (112× magnification, 1 s exposure time for both fluorochromes, FITC and Cy3). Fluorescence signals in the specimens from the transgenic line APOB::GFP were obtained on relaxed alive worms at the same stereomicroscope, and fluorescence intensity was further calculated in ImageJ.

For EdU staining performed on the DV1_8 and DV1_10 worms, microscopy was performed using the confocal microscope OLYMPUS IX83P2ZF on its software, Olympus FluoView™ FV1000 (Olympus, Tokyo, Japan). Z-stack images were adjusted at the same settings, and further specific fluorescent signals from different stacks merged into one layer. The difference in the level of fluorescence signals from dividing cells was calculated using ImageJ.

### 4.7. Statistical Analysis

We generated the boxplots using the online tool Statisty (https//statisty.app). All statistical analyses were performed using R software (version 4.4.2, R Foundation for Statistical Computing, Vienna, Austria). Non-parametric tests were used to calculate differences between worm samples due to unequal sample sizes in the groups. Group comparisons were conducted using the exact Mann–Whitney U test with the wilcox.test function from the stats package. The exact method was specified (exact = TRUE) to compute precise *p*-values without relying on large-sample approximations. Based on the mechanism of RNAi, a one-sided Welch’s *t*-test was applied to evaluate RNAi-induced expression changes, with the directional hypothesis “control > treatment”. Statistical significance in all cases was defined as *p* < 0.05.

## 5. Conclusions

We demonstrated a strong link between four target genes and the homeostasis of *M. lignano*, particularly within its digestive tract. For two genes with known human homologs (*kri1* and *wbp2nl*) our findings support previous predictions of their involvement in transcriptional regulation and high-level biological processes. The other two genes, lacking known homologs, are intriguing candidates as potential regulators or transcription factors specific to *M. lignano*.

Furthermore, the digestive system of *M. lignano* may serve as a valuable model for studying basic mechanisms of regeneration, especially given its possible cell non-autonomous role in neoblast control. The identification of key genes involved in the regulation of feeding and homeostasis in *M. lignano* emphasizes the potential of this model organism to extend our understanding of fundamental biological processes. Future studies focusing on these genes, particularly the *M. lignano*-specific candidates, could unveil novel regulatory pathways and transcription factors unique to free-living flatworms. Additionally, investigation of the role of the intestine in regeneration and metabolic control may provide new insights into neuropeptide signaling and cell non-autonomous mechanisms.

## Figures and Tables

**Table 1:** Morphometry of the *M. lignano* specimens from the DV1_8 and DV1_10 lines before and after RNAi of the target genes. The values represent mean ± 1 SD and include absolute length (L), width (W), area of worm body, area of gonads. N represents the number of measured specimens of *M. lignano*. * marked significant difference (*p* < 0.05). ** not for all worms (one to three individuals) gonads were visually detectable (the details are in Table S4).

Subline/Group	N	L, µm	W, µm	Body Area, µm^2^	Gonads’ Area, µm^2^
DV1_8					
Control	9	1475.5 ±138.8	356.4 ±36.7	530,642.7 ±43,774.7	77,589.1 ±15,892.5
*Mlig-kri1*	22	1205.9 ±151.9 *	297.2 ±44.3 *	405,492.7 ±86,069.3 *	64,158.2 ±19,317.6
*Mlig-tuf1*	24	1242.6 ±114.1 *	253.6 ±39.2 *	383,684.4 ±72,438.6 *	47,225.3 ±14,455.8 *
*Mlig-tuf2*	22	1291.8 ±177.5 *	257.4 ±38.6 *	404,942.7 ±66,601.4 *	45,379.0 ±8668.3 *
*Mlig-wbp2nl*	21	1344.0 ±132.4	263.6 ±31.2 *	433,123.2 ±74,015.0 *	48,747.4 ±12,744.4 *
DV1_10					
Control	9	1560.9 ±101.4	315.6 ±41.9	517,302.3 ±68,565.8	106,516.2 ±27,555.0
*Mlig-kri1*	22	1232.8 ±131.5 *	306.4 ±31.7	436,435.0 ±77,102.0 *	53,749.4 ±11,138.9 *^,^**
*Mlig-tuf1*	22	1190.3 ±110.9 *	301.9 ±34.1	410,264.0 ±39,223.4 *	52,436.7 ±9465.5 *^,^**
*Mlig-tuf2*	20	1156.0 ±157.6 *	298.0 ±50.5	379,303.5 ±68,304.4 *	53,564.6 ±9568.8 *
*Mlig-wbp2nl*	22	1263.2 ±243.8 *	323.1 ±55.2	449,492.7 ±97,078.8	57,707.9 ±10,427.5 *^,^**

## Data Availability

The original contributions presented in this study are included in the article/Supplementary Materials. Further inquiries can be directed to the corresponding author(s).

## Supplementary Materials

The following supporting information can be downloaded at: https://www.mdpi.com/article/10.3390/ijms262411934/s1.

## Abbreviations

The following abbreviations are used in this manuscript:

TFs	Transcriptional factors
APOB	Apolipoprotein B
RNAi	RNA interference
EdU	5-Ethynyl-2′-deoxyuridine (EdU)
dsRNA	double strand RNA
GO	Gene Ontology
