# Supplementary material for: Knockdown-Induced Fasting Phenotypes in Flatworms: Insights into Underlying Mechanisms of Feeding Behavior
Source: Int J Mol Sci. 2025 Dec 11;26(24):11934. doi: 10.3390/ijms262411934 (PMC12732829; doi:10.3390/ijms262411934)

Figure S2. Figure S2. Phalloidin-staining of intact worms in homeostasis (A - L) and the *Mlig-KRI1*(RNAi), *Mlig- TUF1*(RNAi), *Mlig-TUF2*(RNAi), and *Mlig-WBP2NL*(RNAi) worms, subsequently (M - P). Intact worms are presented by hatchlings and juveniles (A - E) with no detectable stylet, seminal vesicles and antrum, and adults (F - H) having the mentioned traits of maturation. Muscle pattern in the RNAi-treated worms is similar to that described for the intact worms.

Due to the peculiarities of sample preparation (fixation and predominantly flattening of worm samples on glass slides), artifacts of local orientation and/or localization of muscle threads are possible: (G - H) muscles around the intestine are displaced laterally; (I - L) fixation at different angles, which sometimes makes it difficult to differentiate the above-mentioned organs in tail, e.g. on image (I) the seminal , the seminal vesicle is quite a small in the tail. Scale bar 300 µm

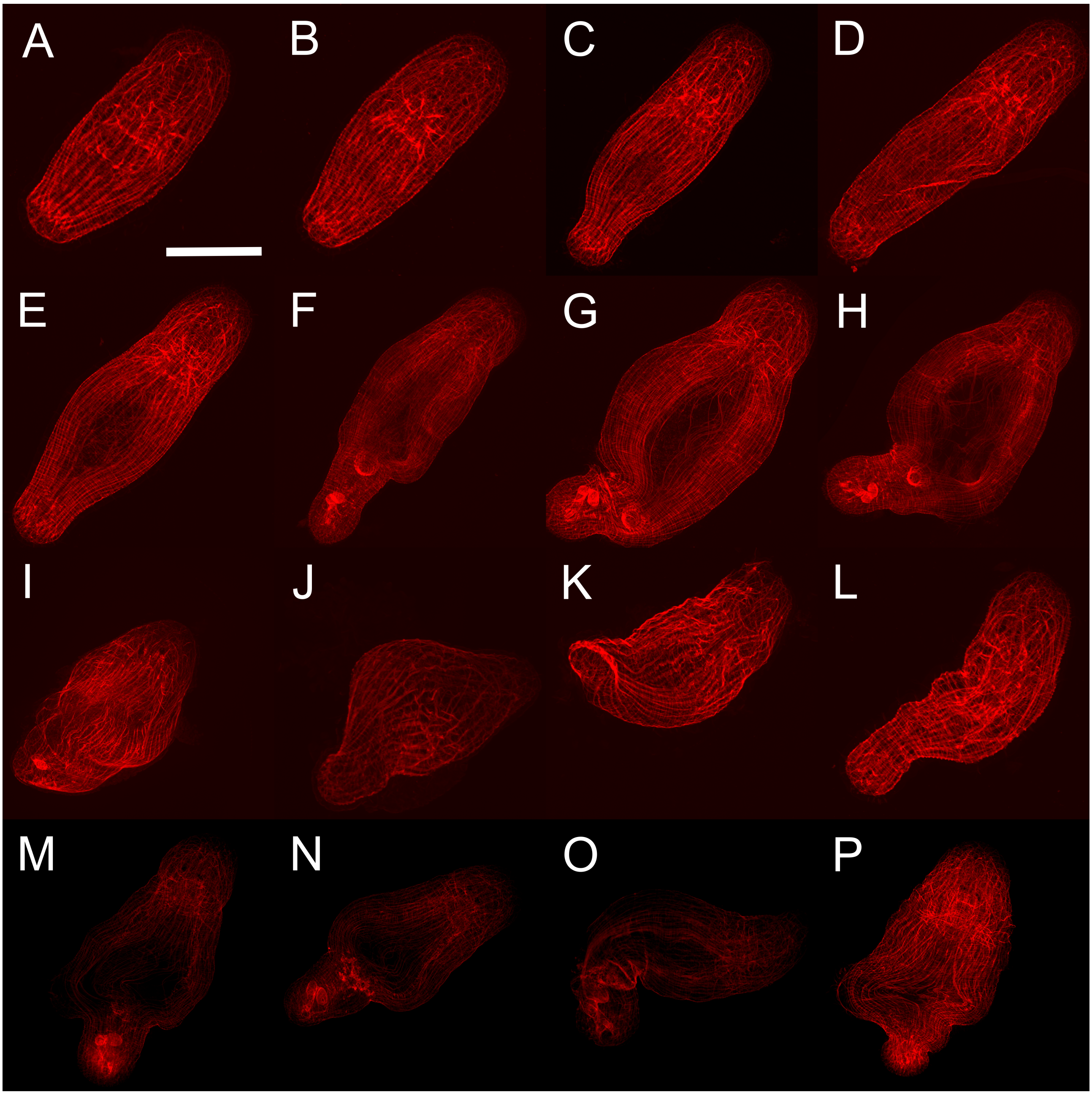

Supplement: Supplementary file 1 [file ijms-26-11934-s001.zip › Figure S2.pdf]
