# Supplementary material for: Knockdown-Induced Fasting Phenotypes in Flatworms: Insights into Underlying Mechanisms of Feeding Behavior
Source: Int J Mol Sci. 2025 Dec 11;26(24):11934. doi: 10.3390/ijms262411934 (PMC12732829; doi:10.3390/ijms262411934)
Supplement: Supplementary file 1 [file ijms-26-11934-s001.zip › Figure S4.pdf]

Figure S4. Results of CD-search requests of *Mlig-TUF1* and *Mlig-TUF2* sequences. For only one protein domain found in *Mlig-TUF1* the tool show all known attributes. For *Mlig-TUF2* sequence where two domain were detected result presents only a structure and a table of domains.

Request for *Mlig-TUF1*

Protein classification: *lipase family protein* (ArchID 10087743)

lipase class 3 family protein may function as a lipase, catalyzing the hydrolysis of ester bonds of insoluble substrates such a triglycerides, or as a feruloyl esterase, hydrolyzing the feruloyl-arabinose ester bond in arabinoxylans and the feruloyl-galactose ester bond in pectin

Attributes:

EC: 3.1.1.- | **PubMed:** 9379943|15081808|20923657|12091482 | **CATH:** 3.40.50.1820 | **SCOP:** 4000732 | **Gene Ontology:** GO:0016788

Viewing Concise H-zoom Hide features

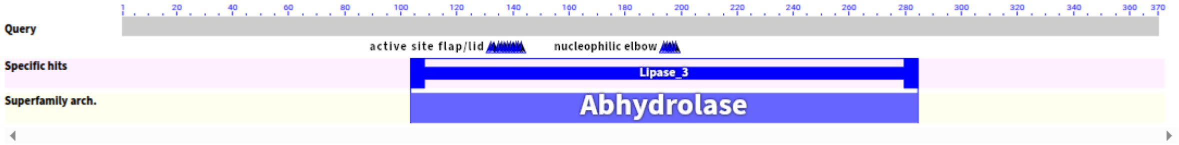

Name	Accession	Description	Interval	E-value
+ Lipase_3	cd00519	Lipase (class 3)	104-284	4.59×10^{-42}

Request for *Mlig-TUF2*

Conserved domains on [local_D5_09c856...]

length=661

Find similar domain architectures

Live BLAST, RID = EC4K66BR014

Database: CDSEARCH/cdd
E-value cut off: 0.01
Composition-based adjustment: yes
Low-complexity filter: no

Refine parameters and search again

Viewing Concise H-zoom Hide features

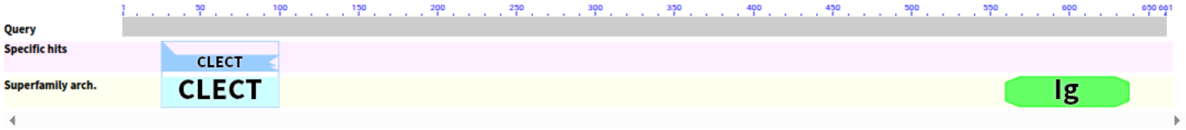

Name	Accession	Description	Interval	E-value
+ CLECT	smart00034	C-type lectin (CTL) or carbohydrate-recognition domain (CRD)	26-99	2.88×10^{-6}
+ IgV_1_Necl_like	cd05717	First (N-terminal) immunoglobulin (Ig)-like domain of the nectin-like molecules; member of the V-set of Ig superfamily (IgSF) domains	560-637	1.41×10^{-4}
