# Supplementary figures and images for: Knockdown-Induced Fasting Phenotypes in Flatworms: Insights into Underlying Mechanisms of Feeding Behavior
Source: Int J Mol Sci. 2025 Dec 11;26(24):11934. doi: 10.3390/ijms262411934 (PMC12732829; doi:10.3390/ijms262411934)

Figure S1. Offspring production per week of experiment in three replicates.

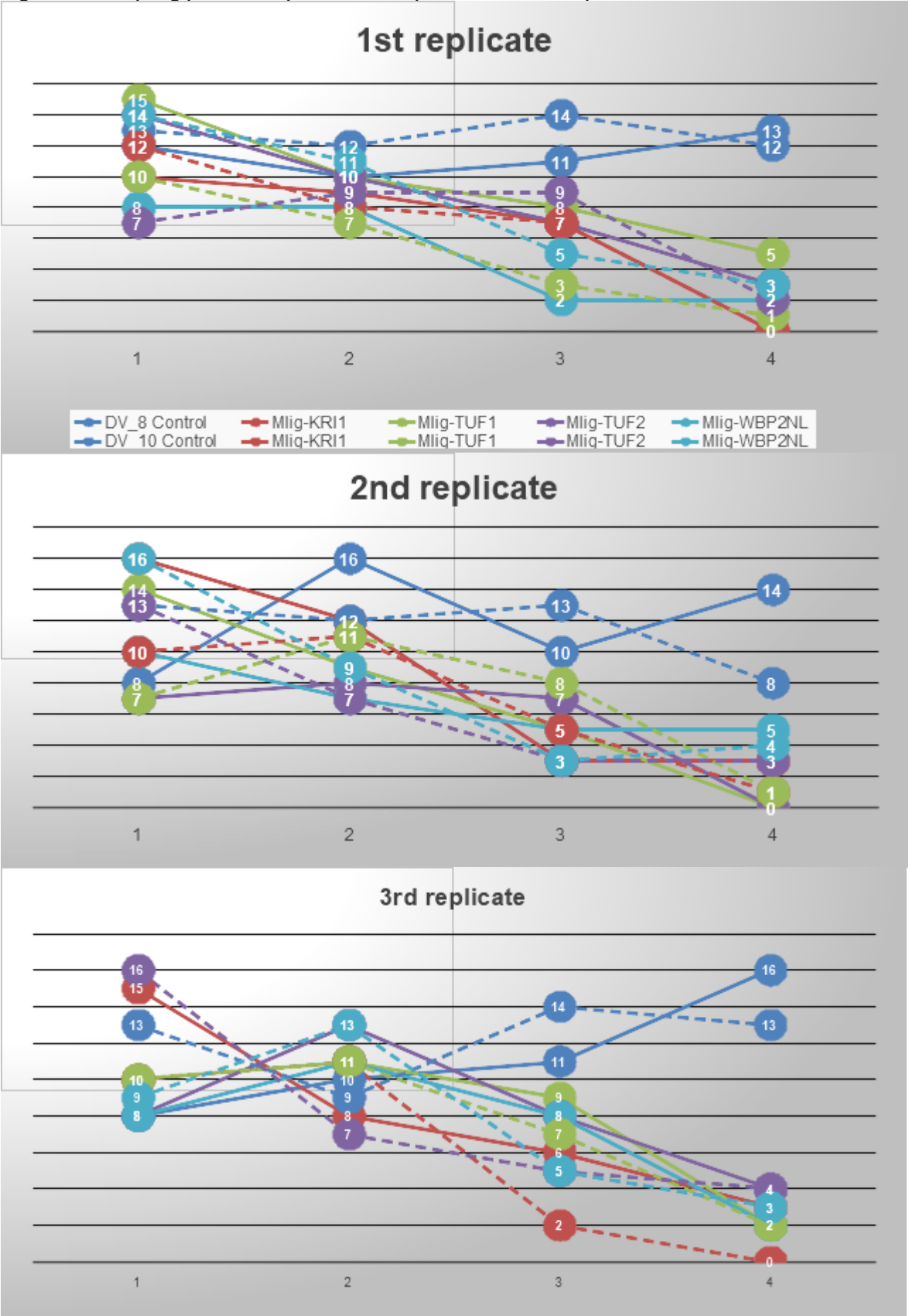

Supplement: Supplementary file 1 [file ijms-26-11934-s001.zip › Figure S1.pdf]

Figure S3. RNAi validation by qPCR.

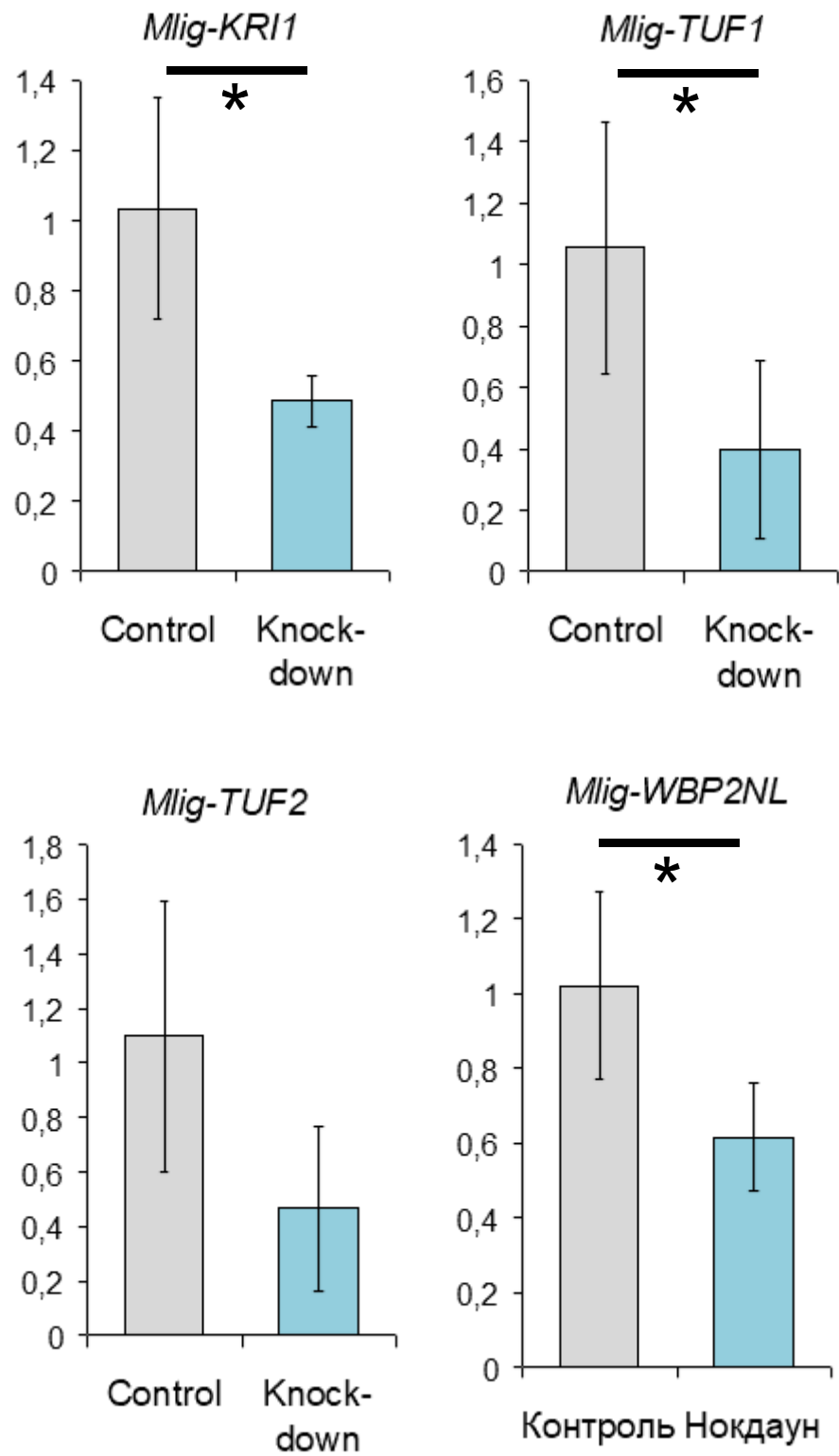

Supplement: Supplementary file 1 [file ijms-26-11934-s001.zip › Figure S3.pdf]
